# Evaluation of low-level laser at auriculotherapy points to reduce postoperative pain in inferior third molar surgery: study protocol for a randomized controlled trial

**DOI:** 10.1186/s13063-016-1540-9

**Published:** 2016-09-02

**Authors:** Hélio Sampaio-Filho, Juliane Sotto-Ramos, Erika Horácio Pinto, Marcia Regina Cabral, Priscila Larcher Longo, Isabel Peixoto Tortamano, Rodrigo Labat Marcos, Daniela Fátima Teixeira Silva, Christine Pavani, Anna Carolina Ratto Tempestini Horliana

**Affiliations:** 1 Postgraduate program in Biophotonics Applied to Health Sciences, Universidade Nove de Julho, UNINOVE, R. Vergueiro, 235/249, CEP 01504-001 São Paulo, Brazil; 2 Integrated Clinic, Department of Estomatology, São Paulo University, São Paulo, Brazil

**Keywords:** Auriculotherapy, Laser, Pain, Postoperative, Third molar surgery

## Abstract

**Background:**

A comfortable postoperative return to daily activities has increased the need to control inflammation after third molar surgery. Anti-inflammatory drugs and analgesics are not exempt from adverse effects such as allergies and chronic gastritis, and they are not without cost. The association between low-level laser and auricular acupuncture can be an alternative when conventional drugs are contraindicated. Among its advantages, we can mention the low risk of side effects, low cost and simplicity of application. The objective of this study is to evaluate the efficiency of low-level laser at auriculotherapy points in reducing postoperative pain in lower third molar surgery.

**Methods/design:**

Ninety bilateral, symmetrical lower third molar surgeries will be performed in 45 healthy patients. Each patient will be their own control, through a split-mouth crossover study. One side of the mouth will be randomly chosen and, immediately after surgery, will be treated with low-level laser. After 21 days, the contralateral side will be operated on with low-level laser simulation used postoperatively. This regimen (laser application or not) will be repeated at 24 and 48 h after surgery. All patients will be requested to take analgesics (acetaminophen) if they have pain, i.e. in case of pain. Neither the surgeon nor the patients will know the assigned treatment. The primary variable will be postoperative pain assessed using a Visual Analog Scale, and the secondary variables will be trismus, edema, local temperature, dysphagia, presence of infection and painkiller ingestion. These variables will be assessed at baseline, 24 h, 48 h and 7 days after surgery. Blood samples for systemic inflammatory cytokine (TNF-α, IL-1, IL-6 and IL-8) analysis will be assessed at baseline and 24 h after surgery.

**Discussion:**

Some authors believe that using a wavelength of 633 to 670 nm is a good option for laser therapy in the field of acupuncture. This wavelength can penetrate biological tissue to a depth of about 3 mm. However, for auriculotherapy points, the stimulus (mustard seeds, needles 1 to 2.5 mm) does not penetrate so deeply. For this reason, we chose a laser wavelength of 660 nm (red wavelength).

**Trial registration:**

ClinicalTrials.gov Identifier: NCT02657174, registered on 11 January 2016.

**Electronic supplementary material:**

The online version of this article (doi:10.1186/s13063-016-1540-9) contains supplementary material, which is available to authorized users.

## Background

Third molar surgery is the most common surgical procedure in dentistry. The postoperative period of third molar surgery is usually accompanied by pain, trismus and swelling, and controlling these symptoms is essential for patient comfort. An inflammatory reaction followed by phlogistic symptoms are common and compromise the quality of life of patients during the first 3 days after surgery [1–3], and about 63 % of patients feel intense pain during the first day after surgery, with greater intensity 3–5 h after the end of anesthesia [4]. Among complications after this type of surgery are alveolar osteitis (dry socket) abscess, fever, reactive lymph node pathology and fever, among others already mentioned [5]. The need for a comfortable postoperative recovery and a rapid return to daily activities has increased the importance of controlling postoperative inflammation, especially with respect to pain and edema. Currently, control of pain and inflammation is conducted with corticosteroids and nonsteroidal anti-inflammatory drugs (NSAIDs) [4, 6, 7]. Anti-inflammatory drugs are often prescribed preventatively and in the postoperative period [4]. However, some studies have shown adverse reactions to NSAIDs, such as gastrointestinal disorders (erosions, ulcers, dyspepsia) with serious bleeding complications, increased cardiovascular complications, kidney failure and platelet alterations [8, 9]. Some studies have discussed the value of auriculotherapy with low-level laser, as well as the benefits of biomodulation in the inflammatory process, working mainly on symptoms such as pain and swelling [10–12]. Some studies have shown that traditional acupuncture is able to interfere with the regulation of both systemic (e.g., interleukin (IL)-10, IL-4) and pro-inflammatory markers (IL-6, IL-1β, tumor necrosis factor alpha (TNF-α)) in some diseases such as asthma and rhinitis [13]. In turn, third molar surgery increases the level of interleukins, especially IL-6, after surgical trauma [14]. This surgical procedure is considered the “gold standard” in pain studies [2, 4] but the behavior of systemic inflammatory markers has not been evaluated after auriculotherapy following third molar extraction.

Acupuncture is an integral part of a system called Traditional Chinese Medicine. According to the World Health Organization (WHO) it stimulates certain points that are distributed throughout the body’s surface using needles, moxibustion, electricity, laser or acupressure [15, 16]. It has been used for many years in several areas of health, but further studies are needed [10, 11, 15, 17–20]. Auriculotherapy or ear acupuncture is one of the acupuncture modalities. Auricular acupuncture can be defined as a system of diagnosis and treatment based on normalization of dysfunctions in the body by stimulating points located on the ear [6, 20], or a therapeutic intervention where stimuli to the external ear are used to relieve health problems in various parts of the body [21]. Auricular therapy can be understood from two perspectives: ancient auricular acupuncture and modern ear acupuncture. The first is based on the stimulation of acupuncture points in the auricle that are connected to the meridians of the body. It is used primarily for the resolution of pain (Chinese School). The second is Paul Nogier’s School, which presupposes a somatotropic organization, representing the whole body in the human ear (European School) [18]. There are several propositions for the theoretical foundations of auriculotherapy. Among them, we can mention the neurological theory, the embryological theory, microsystems, the energy theory of Traditional Chinese Medicine and the hormonal basis [21]. In 1982, the WHO developed a working group aiming to standardize research and clinical uses of auriculotherapy [22]. It intended to establish classification criteria, location and therapeutic value of the points. Despite its efforts, there are still many discrepancies between the two schools described above.

There is only one clinical study to date that evaluates the effect of laser therapy to auricular points for pain reduction in dentistry [23], and there are no randomized controlled clinical trials that validate the effectiveness of auriculotherapy associated with low-level laser to control pain, swelling and postoperative inflammation. In this study, the authors describe a protocol designed to evaluate the application of helium-neon laser or sham to auriculotherapy points after third molar surgery to reduce pain and inflammation.

## Methods/design

The protocol was approved by the Research Ethics Committee of Nove de Julho University (UNINOVE), number #1100869. This study had all necessary consent approved by Nove de Julho University Ethical Committee for any patients who will be involved in the study, including an Informed Consent Form that patients will need to sign to participate. The study is in accordance with the Declaration of Helsinki. Patients who accept participation will sign the Informed Consent Form after verbal (by HSF) and written explanation of the study. The sample will comprise 45 healthy patients of both genders aged from 18 to 28 years who require surgical removal of bilateral symmetrical lower third molars. Patients will undergo two surgeries with a 21-day interval between them in a split-mouth crossover design. The surgeries will be performed by the same surgeon in the Dental Clinic at Nove de Julho University, São Paulo, Brazil, from June 2016 until February 2017.

### Sample size calculation

The sample size was calculated (G* Power software version 3.1.9.2) by a *t* test for paired groups, since we have two groups (right side and left side) on the same patient. The effect size was determined using the formula:

$$ D\kern0.5em =\kern0.5em \frac{Control- Treated}{Standarddeviation}\kern0.5em =\kern0.5em \frac{4.3-2.5}{3.5}\kern0.5em =\kern0.5em 0.51. $$

We select the worst scenario, that is, the largest standard deviation (SD) between means. The mean values of control and treated groups, as well as the SD were taken from one particular study [24]. The error was set at 5 % and the power test at 95 %. According to the calculation, a sample of 45 patients will be necessary to detect differences in pain.

### Inclusion and exclusion criteria

The study will include healthy patients (negative medical history) with their teeth in position IIB according to Pell and Gregory [25], with an indication for the extraction of their third molars (recurrent infections, poor position, orthodontic indication) or professional referral in writing. The following patients will be excluded: those allergic to any drug used in the research (paracetamol (acetaminophen), 2 % chlorhexidine), pregnant or breastfeeding women, smokers, those who have undergone radiotherapy to the head and neck, systemic or local infection (e.g., pericoronitis or periodontal abscess), and those with injuries or radiolucent images associated with the extracted teeth. Patients who have used anti-inflammatory drugs in the last 3 months, and patients who present any complications during surgery (e.g., bleeding, surgical difficulty, surgery longer than 90 min) will also be excluded.

### Randomization

An external researcher, who will not participate in this research (IPT), will perform randomization using Microsoft Excel, 2013 version. As the letters are drawn (A or B), they will be placed into opaque envelopes labeled with sequential numbers. The envelopes will be sealed and remain in numerical order in a safe place until the completion of surgery. The same researcher (IPT) (not involved in the study) will prepare the envelopes. To decide the side of intervention, we will randomize into 45 blocks (1:1) of 90 surgeries designed as A or B. This corresponds to 45 blocks, either AB or BA. As the study will be performed in a double-blind manner, neither the patient nor the operator will know which treatment will be applied in each of the two surgeries. The only person who will know the treatment performed (JSR) will be the researcher who is responsible for the application of the laser to the auriculotherapy points. This data will only be revealed after statistical analysis.

### Design

This is a controlled, randomized, double-blind, split-mouth, crossover clinical trial. In a split-mouth study, a patient functions as their own control:

- G1 – (experimental) 45 third molar surgeries will be performed in a conventional manner [26]. At the end of surgery, low-level laser will be applied to auricular points for prevention of inflammation and pain in the immediate postoperative period and after 24 and 48 h after surgery.
- G2 – (control) 45 surgeries will be performed in the conventional manner, identical to G1. The patient will receive low-level laser, albeit turned off in order to block the passage of light, to the same auricular points used in G1.

The surgeries will be performed with two cartridges (1.8 mL) of the local anesthetic (LA) mepivacaine 2 % with epinephrine 1:1,000,000. The time delay between the surgery on each side will be 21 days.

Acetaminophen (750 mg) 8/8 h will be provided for all patients. They will be instructed to use this only in case of pain, and only for 3 days.

A medical prescription for acetaminophen with codeine phosphate 30 mg (Tylex® Janssen-Cilag) will be provided only in case of severe pain. Patients who need to use this will be excluded from the study. Data from these patients will only be described, but not included in the analysis of results. This exclusion will not jeopardize their treatment for ethical reasons. Any adverse event will be reported in the study. Any important protocol modifications will be communicated.

### Methodology for application of auriculotherapy points

**Fig. 1:**
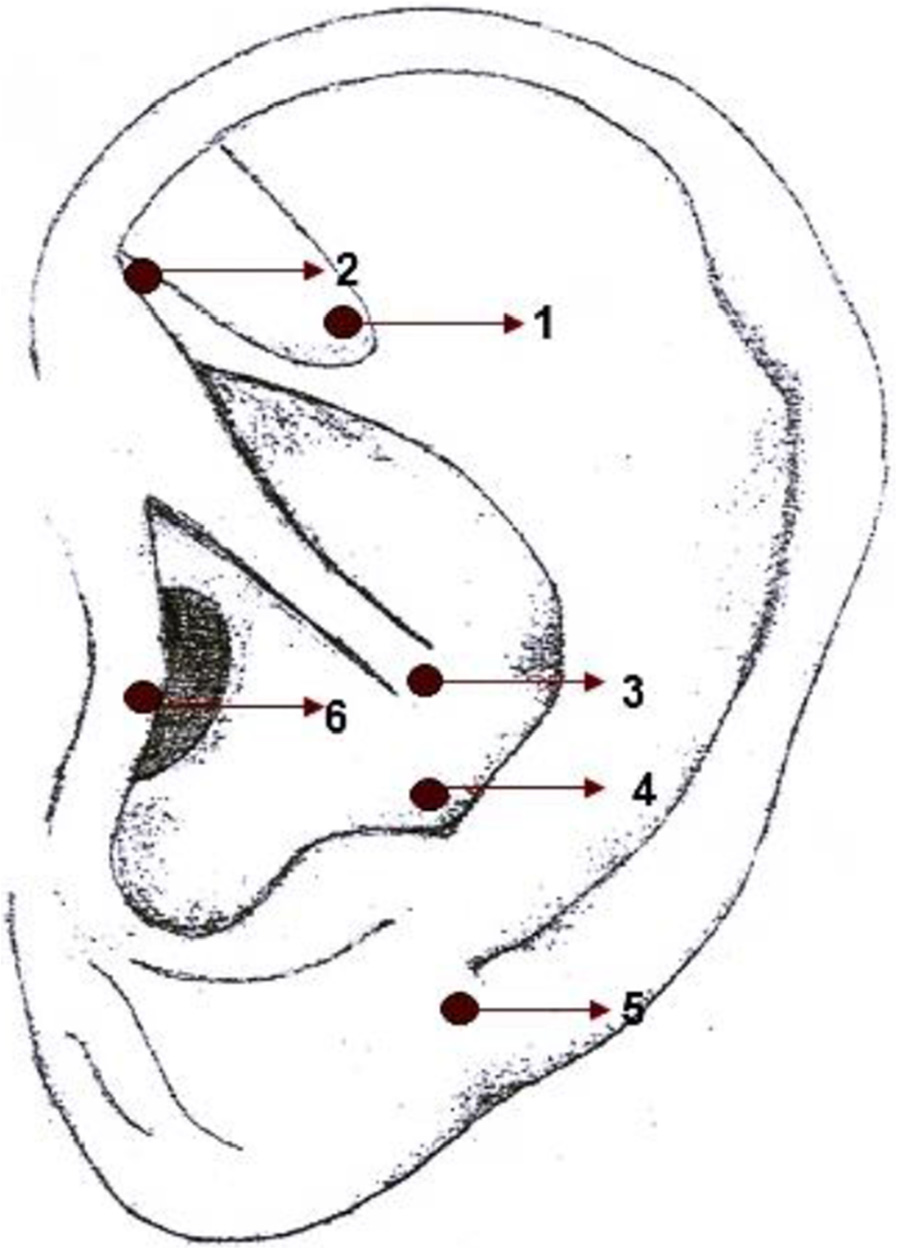
Auricular acupuncture points. Legend: (1) *Shen Men*, (2) Sympathetic (SNV), (3) Stomach, (4) Toothache 3, (5) Jaw, (6) Adrenal

The researcher who will apply the laser will mark the points that will be irradiated on the outer atrium of the patient’s ear with a red gel pen to avoid interference with red laser light (λ = 660 nm). The low-level laser points will be irradiated on the same operated side, based on Oleson [21]; see Fig. 1. The marking of points will be confirmed objectively by a point locator, the Acupoint detector (Acupoint detector MH – II®, Japan) for auriculotherapy, which measures point impedance [27].

### Rationale for the choice of auriculotherapy points

The World Federation of Acupuncture and Moxibustion Societies (WFAS), F7, B Dongjiu Mansion, Xizhaosi street, Dong Cheng District, Beijing. 100061, China. TEL: 86-10-64011210, 10.1186/s13063-016-1540-9 FAX: 86-10-64018354 E-mail: wfas1987@foxmail.com, owing to the fact that there are numerous classifications and auriculotherapy maps used in several countries, has established some norms for the standardization of auricular acupuncture points by bringing together experts who promoted the so-called ISAAPs (International Standard of Auricular Acupuncture Points) [28]. The naming and location was based on subdivisions and models proposed by Oleson [21].

The points to be used in this study are listed below along with their main function: (1) *Shen Men* – relieves pain, anxiety and inflammatory diseases, (2) Sympathetic – SNV – activation by the sympathetic nervous system of parasympathetic sedation, reducing neurovegetative imbalance, (3) Stomach – its related function is to relieve toothache, (4) Toothache 3 – pain relief of the lower teeth, (5) Jaw – relieves pain in the lower teeth, tension and anxiety, and (6) Adrenal (suprarenal) – stimulation of adrenal hormones relieving stress. It is indicated for inflammation and hypersensitivity.

### Low-level laser specifications and dosimetry

The red laser diode (Therapy XT® DMC, São Carlos, Brazil) (ANVISA 80030810157) with a wavelength of 660 nm (±10 nm) will be used. The power of the appliance is 100 mW. The diameter of the fiber optic device is 600 μm and, thus, a spot (area) of 0.002826 cm^2^. The energy delivered per point will be 1 J in 10 s. The radiant exposure will be 354 J/cm^2^ and irradiance will be 35.4 W/cm^2^. In the control side, the device will remain turned off, so neither the patient nor the surgeon will know to which side it is really being applied. We asked DMC (São Carlos, Brazil) to silence the device.

### Study variables

All the variables described below will be evaluated by the same operator at baseline, and 24 h, 48 h and 7 days after each surgery. The primary outcome will be postoperative pain assessed by a Visual Analog Scale (VAS). Secondary outcomes will be trismus, edema, local temperature, dysphagia, and presence of infection (systemic temperature, lymphadenopathy).

The pain will be assessed by applying a VAS, consisting of a 100-mm line numbered in centimeters, with two closed ends. One end is labeled “0” and the other “100,” meaning “no pain” and “worst pain already felt,” respectively. Each patient will be instructed to mark a vertical line with the point that best matches the intensity of pain during the evaluation. Instructions on marking will always be given to the patient by the same operator [29]. These assessments will be done at baseline, and 24 and 48 h after surgery. The opposite side of the mouth will be evaluate identically but after a 21-day interval.

**Fig. 2:**
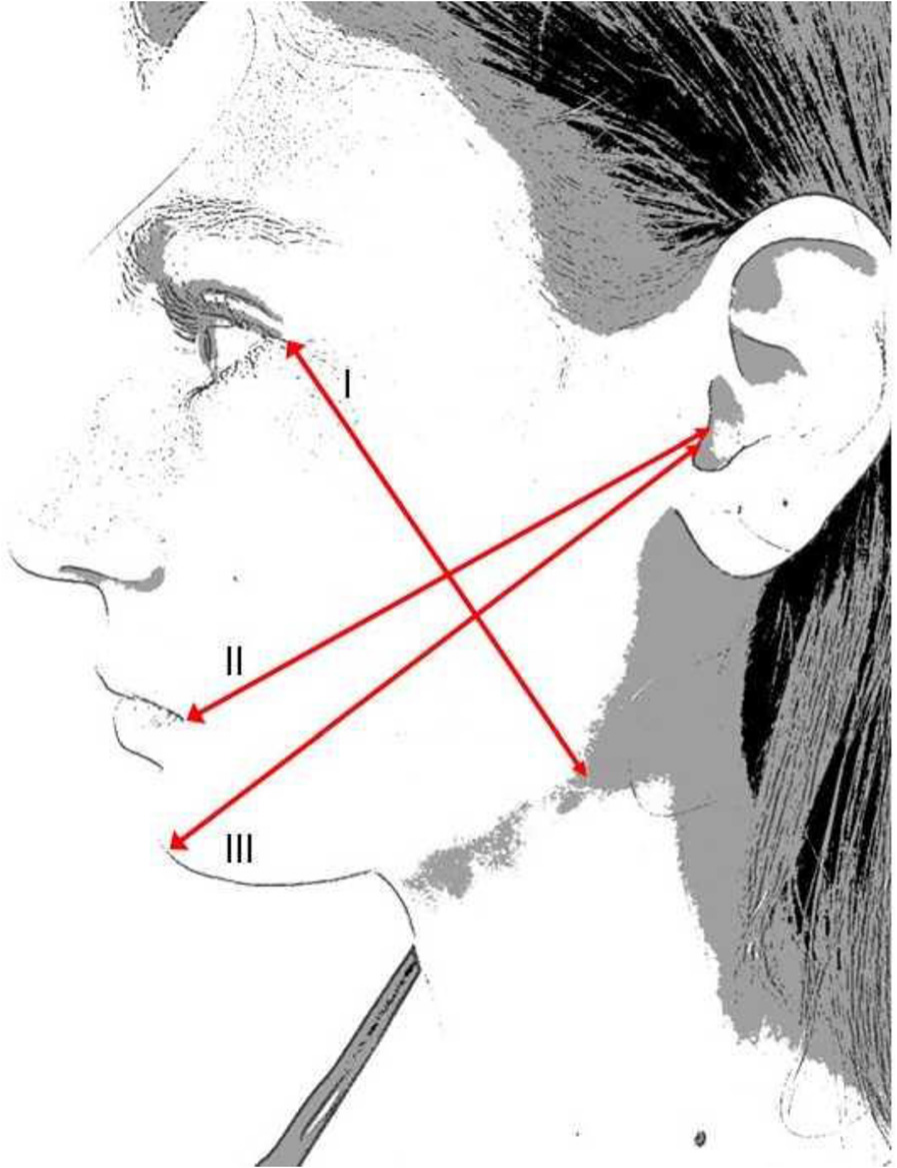
The criteria for the determination of edema. Legend: (*I*) The corner of the eye to the angle of the jaw, (*II*) The tragus to the labial commissure, and (*III*) The tragus to pogonion

The criteria for the determination of edema will follow preestablished measurements: (1) the corner of the eye to the angle of the jaw, (2) the tragus to the labial commissure, and (3) the tragus to the pogonion [30]; see Fig. 2.

For the evaluation of the presence of trismus the inter-incisor measurement (distance between the incisal edge of the maxillary central incisor and lower) will be used with a digital caliper (Mitutoyo Digimatic Caliper model, Kawasaki, Japan). During clinical examination (7 days before the surgery), the patient will be asked to perform their maximum mouth opening, which will be measured in millimeters [31].

The local temperature will be measured in the mandibular angle region 2 cm above the lower jaw board (Safety 1st® model “No Touch Forehead”, Columbus, OH, USA).

Fever (systemic) is usually an indicator of infection. Since, in case of infection pain is usually worse, this may become a bias. The temperature will be measured using a digital thermometer (Safety 1st® model “No Touch Forehead”, Columbus, OH, USA).

The assessment of dysphagia will be conducted through a numerical scale: 0 – indicates total absence of dysphagia; 1 – dysphagia for solid food; 2 – dysphagia for any food, liquid or solid.

**Fig. 3:**
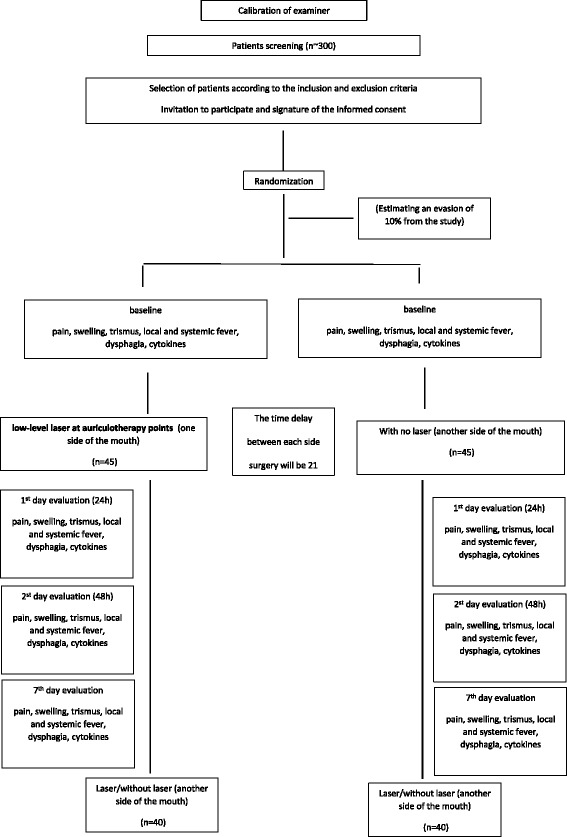
Flowchart presenting an overview of the study

Rescue medication intake – the quantity of medicine ingested will be counted and statistically analyzed. All patients will be advised to take only acetaminophen in case of pain. We will ask for tablet return in order to monitor adherence to intervention protocols. A flowchart is presented for an overview of the study (Fig. 3)

**Fig. 4:**
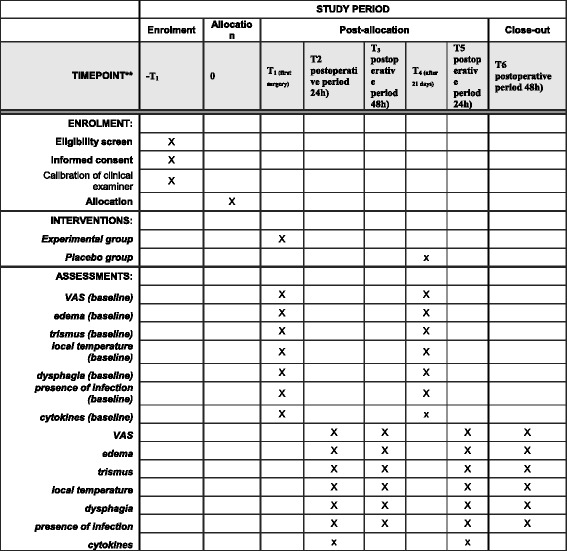
Template of recommended content for the schedule of enrollment, interventions and assessments.* Legend: *Recommended content can be displayed using various schematic formats. See SPIRIT 2013 Explanation and Elaboration for examples from protocols (Additional file 1)

### Profile of plasma and salivary cytokines

Blood samples will be collected in dry tubes and sent to the laboratory of Biophotonics Applied to Health Sciences (Nove de Julho University). After centrifugation, serum will be stored in 1.5-mL tubes at −80 °C. Plasma levels of inflammatory markers of TNF-α, IL-1, IL-6 and IL-8 will be quantified using commercial ELISA kits (Peprotech Inc., Rocky Hill, NJ, USA) according to the manufacturer’s instructions.

### Statistical analysis

The data will be tabulated on an Excel table developed for research.

By the end of the study, randomized groups will be revealed and statistical analysis will be performed as group A or group B. The meaning of A or B will be known after statistical analysis. The data will be evaluated by comparing the two groups: experimental and control groups (“gold standard”). Data will be submitted to the Shapiro-Wilk test to test for data normality. If the data is normal a homogeneous *t* test will be applied. For nonparametric data and scores the Mann-Whitney test will be applied. Data will be presented as means ± SD and the *p* value set at 0.05.

## Discussion

This study aims to evaluate the effectiveness of auriculotherapy stimulation with laser on postoperative pain in patients undergoing third molar surgery. The main rationale for conducting this study is for the reduction of postoperative anti-inflammatory drug and analgesic use because of the frequency of adverse effects associated with their use [8, 9]. Extraction of third molars is considered the best model for assessing acute pain [32] and has been used for a long time [33]. To avoid bias, the two surgeries (experimental side and control side) should be performed as closely as possible; however, a time interval between surgeries is necessary to avoid distortions in patient’s judgement of their pain [34]. Therefore, it is important to have control variables: surgical technique, tooth position and variables inherent to the surgeon. Additionally, the surgical technique should be standardized as much as possible in order to cause comparable surgical trauma. The incision should also be standardized, as should the osteotomy and odontosection.

Some specific techniques are proposed to evaluate tooth position. The Winter and the Pell and Gregory techniques are the most common. Despite the Winter technique being simpler and more easily reproducible [35], it does not consider adequate parameters for the standardization of symmetry between the sides. The Pell and Gregory technique is more complete [25]. It evaluates the tooth depth position in the vertical direction (relative to the occlusal plane and in relation to the neck of the adjacent second molar) and the horizontal (as it evaluates the position of the tooth in relation to the ramus of the mandible). In order to classify the position of a tooth, a panoramic radiograph is usually sufficient. Teeth should be as symmetrical as possible. The variables inherent to the surgeon, such as length of professional experience, manual dexterity, and level of training, can be controlled when only a particular surgeon performs all surgeries. Moreover, the split-mouth technique allows the patient to be their own control, thus reducing variation between individuals. In this case, it eliminates factors such as subjectivity of pain, inflammatory response, degree of mouth opening, elasticity of the buccal mucosa, size of the oral rhyme and anatomical variations between the experimental and control groups, because the experimental and placebo treatments are performed on the same person. Also, regarding the similarity of procedures to minimize differences between interventions, the device will remain connected on one side and turned off on the control side, so that neither the patient nor the surgeon will know to which side it is really being applied. This is an important issue because the device emits sound during its operation. Because of this, we asked the company which manufactures the laser to silence the device. For its use, one researcher will apply the laser, and yet another one will measure the variables (pain, trismus, edema, local temperature, dysphagia and presence of infection by means of systemic temperature measurement and assessing the degree of lymphadenopathy).

A red diode laser with a wavelength of 660 nm (±10 nm) will be used. It has been proved that a wavelength of 650 to 950 nm can penetrate biological tissue to a depth of 3 mm [36]. However, some authors believe that a wavelength of 633 to 670 nm is the best option for laser therapy [15]. For acupuncture, it would be necessary to use a wavelength that achieves greater depth, since the acupuncture needle is 15 to 70 mm long, thus in this case a 810-nm (near-infrared) laser would be more appropriate. However, for auriculotherapy points, the stimulus (mustard seeds, needles 1 to 2.5 mm) does not penetrate tissue so deeply, so the 660-nm laser (red wavelength) seems sufficient for our study. For this reason, we chose the lower wavelength 660-nm laser.

It has been shown that the irradiance required to achieve the same effect as an acupuncture needle must be greater than 1.3 W/cm^2^ [37]. Other authors indicate radiant exposure of 0.001 J/cm^2^ to 10 J/cm^2^ or more [15]. One of the reasons for choosing auriculotherapy is for convenience of application as with it there is no need for the introduction of an intra-appliance tip orally (Pages-Escobar, 2010; [2, 38]).

The auricular points were chosen based on the Chinese School [18], as suggested by Oleson [21]. This study followed the Standards for Reporting Interventions in Clinical Trials of Acupuncture (STRICTA) recommendations, a new, specific protocol for acupuncture studies [39].

Postoperative pain will be our primary variable, so it is very important to measure it properly. Patients will always take the same medication regardless of which side has been operated on. Acetaminophen will be given to patients because it is a safe and potent analgesic for acute soft tissue pain, showing no inferiority when compared with NSAIDs [40]. Although the mechanism of action of acetaminophen has not yet been fully elucidated, it is known that, as with NSAIDs, it is sufficient for peripheral pain [41]. The selection of acetaminophen was based in the parameters recommended by the WHO [42]. All patients will be advised to take only acetaminophen in case of pain. At the relevant moment, they will be encouraged to write down the day, time and pain intensity at the time they decided to take the medication. In case of severe pain, they will be instructed to contact the surgeon and begin a regimen of codeine plus acetaminophen. If the parameters for selecting the appropriate analgesics [42] is are not adhered to, the effect of the treatment could be masked, and could bias the data, as reported in other studies [31].

For pain measurement, a VAS will be included as the main instrument for assessing the patients’ experience of pain. This scale is graduated in centimeters, which allows the use of parametric tests, which in turn improves accuracy of data analysis. Infection parameters (systemic disease) will also be evaluated. Such patients will be excluded from the study, but accounted for and described according to the CONSORT statement. The anti-inflammatory drugs and analgesics have been classically used for treating pain after third molar surgeries, but they are not exempt from adverse effects, such as allergies and chronic gastritis. The association between low-level laser and auricular acupuncture may be a low-risk alternative to abolish the need for, or reduce the quantity of, these postoperative medications.

### Trial status section

This trial has not initiated patient recruitment at the time of submission.

## Additional file

Additional file 1: SPIRIT 2013 Checklist: Recommended items to address in a clinical trial protocol and related documents* (DOC 123 kb)

## Abbreviations

IL: Interleukin
ISAAPs: International Standard of Auricular Acupuncture Points
LA: Local anesthetic
NSAID: Nonsteroidal anti-inflammatory drug
TNF-α: Tumor necrosis factor alpha
VAS: Visual Analog Scale
WFAS: World Federation of Acupuncture-Moxibustion Societies
WHO: World Health Organization
